# Comparison of *in situ* ruminal straw fiber degradation and bacterial community between buffalo and Holstein fed with high-roughage diet

**DOI:** 10.3389/fmicb.2022.1079056

**Published:** 2023-01-09

**Authors:** Xuan Xuan Pu, Xiu Min Zhang, Qiu Shuang Li, Rong Wang, Min Zhang, Shi Zhe Zhang, Bo Lin, Bie Tan, Zhi Liang Tan, Min Wang

**Affiliations:** ^1^Department of Animal Science and Technology, University of Hunan Agricultural University, Changsha, Hunan, China; ^2^CAS Key Laboratory for Agro-Ecological Processes in Subtropical Region, National Engineering Laboratory for Pollution Control and Waste Utilization in Livestock and Poultry Production, Institute of Subtropical Agriculture, The Chinese Academy of Sciences, Changsha, Hunan, China; ^3^Department of Animal Science and Technology, University of Guangxi, Nanning, Guangxi, China

**Keywords:** rumen ecosystem, straw fiber degradation, bacterial community, *in situ*, buffalo

## Abstract

Buffalo exhibits great efficiency in utilizing low-quality roughage, which can be due to the combined effect of host physiological feature and roughage diet fed. The present study was designed to compare the ruminal fiber degradation and the bacterial community attached to straws in buffalo and Holstein when fed with the same high-roughage diet using *in situ* ruminal incubation technique. Rice and wheat straws were selected as the incubation substrates and sampled at 0, 4, 12, 24, 48, 72, 120, and 216 h of incubation time to measure the kinetics of dry matter (DM) and neutral detergent fiber (NDF) disappearance. Additional two bags were incubated and sampled at 4 and 48 h of incubation time to evaluate the bacterial community attached to straws. The results showed that buffalo exhibited a greater (*p* ≤ 0.05) fraction of rapidly soluble and washout nutrients and effective ruminal disappearance for both DM and NDF of straw than Holstein, together with a greater (*p* ≤ 0.05) disappearance rate of potentially degradable nutrient fraction for NDF. Principal coordinate analysis indicated that both host and incubation time altered the bacterial communities attached to straws. Buffalo exhibited greater (*p* ≤ 0.05) 16S rRNA gene copies of bacteria and greater (*p* ≤ 0.05) relative abundance of *Ruminococcus* attached to straw than Holstein. Prolonging incubation time increased (*p* ≤ 0.05) the 16S rRNA gene copies of bacteria, and the relative abundance of phyla Proteobacteria and Fibrobacters by comparing 4 vs. 48 h of incubation time. In summary, buffalo exhibits greater ruminal fiber degradation than Holstein through increasing bacterial population and enriching *Ruminococcus*, while prolonging incubation time facilitates fiber degradation through enriching phyla Proteobacteria and Fibrobacteres.

## Introduction

Buffalo is considered to be the livestock with immersed value to be developed, providing economic value from meat, milk, leather production, and draft power for rice cultivation for thousands of years (Tong et al., 2022). Buffalo usually lives in tropical and subtropical area zones that are characterized by elevated temperatures and low-quality roughage with high levels of structural carbohydrates, such as cereal straws, tree foliage, and brown sugar residues. Buffalo exhibits better adaptation to hot and humid tropics than other cattle for their greater resistance to heat, certain diseases, and pests. In addition, buffalo also have a greater ability to digest roughage, especially when the roughage is of low quality (Hussain and Cheeke, 1996; Xu et al., 2021).

The rumen is a fermentation ecosystem with strong fiber lysis, as it contains a wide microbial community of bacteria, protozoa, archaea, fungi, and bacteriophages that rapidly colonize and digest plant materials in a complex and coordinated manner (Stevens and Hume, 1998; Kamra, 2005; Mizrahi et al., 2021). The end products of rumen fermentation are short-chain fatty acids and methane, which form a major metabolic fuel for host animals and contribute to anthropogenic greenhouse gases, respectively (Beauchemin et al., 2020; Wang et al., 2021, 2022). It has been widely reported that buffalo has a distinct rumen microbial community through enriching cellulolytic bacteria (Wanapat et al., 2000; Chanthakhoun et al., 2012), leading to greater fiber digestibility of roughage. However, the dynamics of fiber degradation in the rumen and the colonization of bacteria on the roughage were rarely studied together to explore the mechanism of difference in fiber degradation capacity between buffalo and other cattle. In addition, the unique microbial community in buffalo can be driven by both the host and diet (Fernando et al., 2010; Henderson et al., 2015; Li et al., 2019). Few studies have been conducted to investigate the dynamic changes of fiber degradation and bacterial community in buffalo by comparing them with other cattle under the same high-roughage feeding strategy.

The nylon bag technique has been developed in the 1930s and widely employed to quantify the ruminal degradation of feed in ruminants. The nylon bag technique exhibits good repeatability without the influence of physiological conditions of host animals and begins to attract great interest to study associations between the kinetics of feed degradation and the bacterial community attached to the rumen microbial ecosystem (Cheng et al., 2017; Zhang et al., 2020). The present study was designed to employ this technique to investigate whether rumen bacteria of buffalo could exhibit a greater fiber digestibility of straw by comparing with Holstein when fed with the same high-roughage diet. We then measured the kinetics of DM and NDF disappearance of two straws (i.e., rice and wheat straw) through 216 h of incubation (Zhang et al., 2020) and explored the bacterial community attached to straw at 4 and 48 h of incubation time through 16S rRNA gene amplicon sequences.

## Materials and methods

The experiment was conducted in Guangxi Taiminxing Animal Husbandry Co. Ltd., Nanning, China. The experimental procedure was approved by the Animal Care Committee, Institute of Subtropical Agriculture, the Chinese Academy of Sciences, Changsha, China.

### Animals

Three buffalo (218 ± 13.11 kg) and three Holstein (280.33 ± 7.51 kg) fitted with custom rumen cannula (101.3 mm internal diameter) were randomly employed to conduct the *in situ* ruminal incubation experiment. All cattle were fed two times per day at 08:00 and 16:30 with a total mixed diet containing 20% of concentrate and 80% of the wheat straw mixture (Supplementary Table S1) and had free access to fresh water. The rumen content was collected through a rumen cannula before morning feeding after a 30-day adaptation period of the diet and stored at −80°C for microbial DNA extraction to analyze the bacterial community of each animal.

### Substrate preparation

Two substrates, including rice and wheat straw, were collected and ground to pass a 4-mm screen using a Wiley mill with a standard sieve (450 μm porosity, Shaoxing Shangyu Shengchao Instrument Equipment Co., Ltd., Shaoxing, China). The chemical compositions of substrates are listed in Table 1. For each animal, a total of 18 bags (10 cm × 20 cm, 40 μm porosity; Shanghai Yanjin Hardware Electromechanical Operation Department, Shanghai, China) containing 6.5 g of each substrate were prepared with 16 bags used for kinetics of fiber disappearance and two bags used for changes in the bacterial community attached to straw particles.

**Table 1:** Chemical composition of rice and wheat straw (g/kg DM).

Items	Straw	
	Wheat	Rice
OM	914	894
CP	30.3	62.3
NDF	756	704
ADF	458	374

OM, organic matter; CP, crude protein; NDF, neutral detergent fiber; ADF, acid detergent fiber.

### *In situ* ruminal incubation and sampling

The bags were placed inside large mesh bags (40 cm × 40 cm) with three compartments to prevent them from aggregating together and incubated in each animal for 0, 4, 12, 24, 48, 72, 120, and 216 h in a staggered manner to ensure no more than three mesh bags in the rumen. Additional two bags were incubated for 4 and 48 h to evaluate the bacterial community attached to straws. When the incubation was ended, nylon bags were removed from the rumen and put into ice water to terminate microbial disappearance. The bags were then washed, dried at 105°C, weighed, and stored until measuring fiber content.

Bags for bacterial community analysis were gently washed with phosphate-buffered saline (pH = 7.4) to remove the ruminal contents and loosely adhered microbes from the outer surface of the bag. Then, the liquid in the bags was gently squeezed to extract the liquid which was then discarded. The residues were removed from the bags and transferred into 15-ml tubes, flash-frozen in liquid N_2_, frozen vacuum-dried, and stored at −80°C until microbial DNA extraction.

### Chemical analysis

All samples of substrates were dried and ground to pass through a 4-mm sieve. The DM (method 945.15), organic matter (OM, method 942.05), and crude protein (CP, method 954.01) were measured according to Association of Official Analytical Chemists (AOAC) (2005). NDF was first analyzed by using a neutral detergent solution without the addition of sodium sulfite and α-amylase, and acid detergent fiber (ADF) was then analyzed using an acid detergent solution according to Van Soest et al. (1991).

### Microbial analysis

#### DNA extraction

About 0.50 mL of ruminal fluid or 0.20 g of frozen-dried samples from nylon bags was used to extract microbial DNA using a modified RBB + C methodology (Yu and Morrison, 2004) with sand beating according to Ma et al. (2020). The quality of the DNA extracts was assessed using agarose gel (1%) electrophoresis. The concentrations of total DNA extracted were measured using an ND-2000 spectrophotometer (NanoDrop Technologies, Wilmington, DE) and then stored at −80°C until further analyses.

#### Quantitative real-time PCR analyses

The procedures of qPCR were followed by Jiao et al. (2014) and Ma et al. (2018). Selected groups of microorganisms included bacteria, *Fibrobacter succinogenes*, *Selenomonas ruminantium*, *Prevotella* spp., and *Ruminococcus amylophilus* with primers (Supplementary Table S2). All samples were assayed in double. The PCR efficiency (E) for all the standard curves was calculated by the slope of the standard curve: E = 10^(−slope)-1^. Final absolute amounts of target groups or species were estimated by relating the C_T_ value to the standard curves and expressed as log_10_ copies/DM contents.

#### 16S rRNA sequencing and analysis

The amplicon sequencing and analyzing were performed at the MiSeq platform through Illumina sequencing at Shanghai Biozeron Biological Technology Co. Ltd. according to our previous study (Li et al., 2022). Briefly, extracted purified DNA (10 ng/μL) from each rumen sample was subjected to PCR amplification of the V3-V4 region of 16S rRNA gene using universal bacterial primers 341F: (5′-CCTAYGGGRBGCASCAG-3′) and 806R: (5′-GGACTACNNGGGTATCTAAT-3′) according to Zakrzewski et al. (2012). After PCR amplification, all amplicon libraries were sequenced, and the barcodes and sequencing primers were removed before data processing. The passed sequences were dereplicated and subjected to the DADA2 algorithm to identify indel mutations and substitutions, which resolves amplicon sequence errors to generate amplicon sequence variants (ASVs). The phylogenetic affiliation of each 16S rRNA gene sequence was analyzed by RDP Classifier^1^ against the silva (SSU138) 16S rRNA database using a confidence threshold of 70% (Amato et al., 2013; Callahan et al., 2016). The rarefaction analysis based on Mothur v.1.21.1 (Schloss et al., 2009) was conducted to reveal the alpha diversity, including Chao1, Shannon’s diversity, number of observed ASV, and taxonomic abundance. Beta diversity was analyzed based on Bray–Curtis similarity distances (Bray and Curtis, 1957) and was performed using Mothur v 1.41.1 according to the instruction.

#### Calculations and statistical analysis

The exponential model of Ørskov and McDonald (1979) was used to fit the kinetics of *in situ* ruminal nutrient disappearance:


$$p=a+b\times \left(1-e^{\left(-c\times t\right)}\right)$$

where *p* is the nutrient disappearance at time *t* (g/kg); *a* is the fraction of rapidly soluble and washout nutrient (g/kg); *b* is the fraction of potentially degradable nutrient (g/kg); *c* is disappearance rate (%/h) of fraction *b*. Data were fitted using the Nonlinear Regression Analysis Program (NLREG, version 5.4).

The effective ruminal degradation (*ED*, g/kg) was calculated according to the equation described by McDonald (1981) as follows:


$$ED=a+b\times c/\left(c+k\right)$$

where *k* was the passage rate from the rumen (h^−1^) and is set to 0.02 h^−1^ and 0.06 h^−1^ in the present study.

Data were analyzed using SPSS 26.0 software (Chicago, IL, United States). The first model for analyzing the disappearance of straws was a linear mixed model, which included animal types (*n* = 2) and straws (*n* = 2) as the fixed effect. When two sampling times were included, the second model included animal type (*n* = 2) as a fixed effect, sampling time (*n* = 2) as a repeated measurement, and animals as the random effect (*n* = 3). The interaction effect was excluded from the analytic models for the lack of significant effect (*p* > 0.05). Significance was considered when *p* ≤ 0.05, and the tendency of significance was considered when 0.05 < *p* ≤ 0.1.

## Results

**Figure 1:**
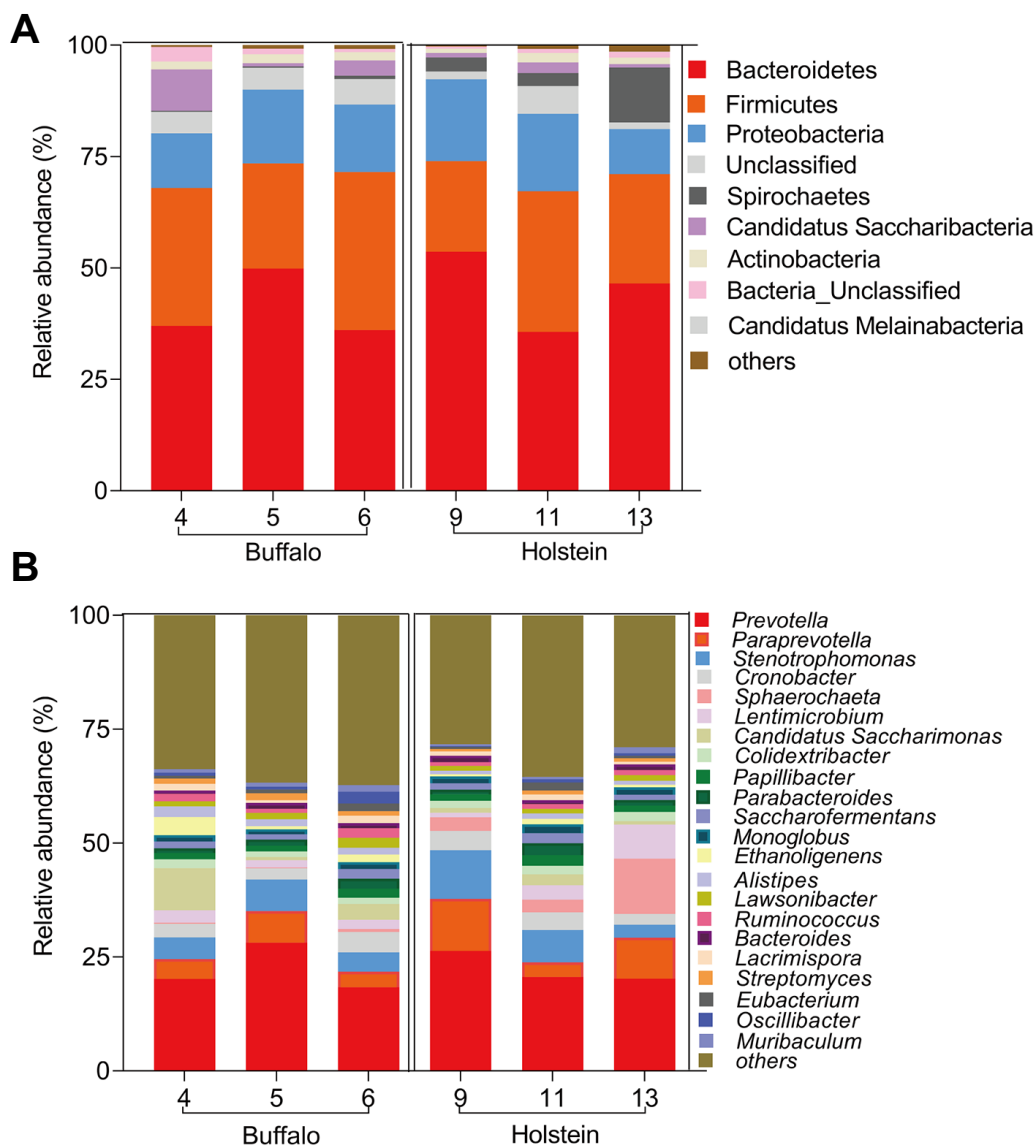
Bacterial community in buffalo or Holstein rumen microbiome at phylum **(A)** and genus level **(B)**. Numbers 4, 5, and 6 represent the ear tags of buffalo, while numbers 9, 11, and 13 represent the ear tags of Holstein.

The rumen samples were first collected and analyzed through 16S rRNA sequencing technique to provide the ruminal bacterial composition of buffalo or Holstein fed with the same high-roughage diet. The main dominant ruminal phyla were Bacteroidetes, Firmicutes, and Proteobacteria, and the dominant genera were *Prevotella*, *Paraprevotella*, *Stenotrophomonas,* and *Sphaerochaeta* in both buffalo and Holstein (Figure 1).

The nutrient degradation kinetics was investigated by the *in situ* ruminal incubation technique (Table 2). Buffalo generally exhibited greater (*p* ≤ 0.05) *a*, *ED_2,_* and *ED_6_* for both DM and NDF disappearance, greater (*p* ≤ 0.05) *c* of NDF disappearance (*p* ≤ 0.05), and a tendency of greater *c* of DM disappearance (*p* ≤ 0.1) of straw than Holstein. Furthermore, rice straw had greater (*p* ≤ 0.05) values of *a* + *b*, *ED_2_,* and *ED_6_* and lower (*p* ≤ 0.05) values of *a* for DM and NDF disappearance than wheat straw. Both host and incubation time altered the 16S rRNA gene copies of bacteria (Table 3). Buffalo had greater (*p* ≤ 0.05) 16S rRNA gene copies of bacteria attached to straw particles when compared with Holstein. The incubation time of 48 h had greater (*p* ≤ 0.01) 16S rRNA gene copies of bacteria attached to straw particles than 4 h. Wheat straw had greater (*p* ≤ 0.05) 16S rRNA gene copies of *Fibrobacter succinogenes* than rice straw.

**Table 2:** Characteristics of DM and NDF disappearance of wheat and rice straws after 216 h of *in situ* ruminal incubation time in buffalo and Holstein rumen.

Items^a^	Cattle		Straw		SEM	Value of *p*	
	Holstein	Buffalo	Wheat	Rice		Cattle	Straw
DM							
*a*	50.2	69.5	69.9	49.8	0.34	<0.01	0.02
*b*	644	632	597	679	1.00	<0.01	0.40
*c*	1.90	2.30	2.00	2.20	0.001	0.07	0.09
*a* + *b*	694	701	667	728	0.88	0.56	<0.01
ED_2_	361	408	366	404	0.63	0.02	<0.01
ED_6_	204	245	218	231	0.69	<0.01	0.02
NDF							
*a*	73.6	90.1	90.6	73.1	0.18	<0.01	<0.01
*b*	617	617	572	663	0.72	0.99	<0.01
*c*	1.93	2.27	2.02	2.18	0.10	0.03	0.23
*a* + *b*	691	708	662	736	0.76	0.15	<0.01
ED_2_	377	417	375	419	0.43	<0.01	<0.01
ED_6_	224	259	233	250	0.48	<0.01	<0.01

a *a*, the intercept of the disappearance curve at 0 h representing the very rapidly degradable and washout fraction of nutrients (g/kg); *b*, the potential degradation of nutrients representing the more slowly degraded component (g/kg); *c*, the rate constant for the degradation of b (10^−2^/h); ED_2_ and ED_6_: effective ruminal degradation of nutrient with ruminal passage rate set at 0.02 h^−1^ and 0.06 h^−1^, respectively.

DM, dry matter; NDF, neutral detergent fiber; SEM, standard error of mean.

**Table 3:** Selected microbial groups (log_10_ gene copies per g rumen content) attached to rice and wheat straws at 4 and 48 h of *in situ* incubation time in buffalo and Holstein rumen.

Items	Cattle		Time (h)		Straw		SEM	Value of *p*		
	Holstein	Buffalo	4	48	Wheat	Rice		Cattle	Time	Straw
Bacteria	12.3	12.6	12.3	12.7	12.6	12.4	0.17	0.03	<0.01	0.08
*Fibrobacter succinogenes*	9.54	9.92	9.73	9.73	10.3	9.20	0.37	0.17	0.99	<0.01
*Selenomonas ruminantium*	6.91	6.90	6.88	6.93	6.90	6.92	0.12	0.92	0.32	0.73
*Prevotella* spp.	11.4	11.7	11.5	11.6	11.6	11.5	0.14	0.15	0.94	0.10
*Ruminococcus amylophilus*	8.73	8.75	8.89	8.59	8.66	8.83	0.24	0.91	0.10	0.33

SEM, standard error of mean.

**Figure 2:**
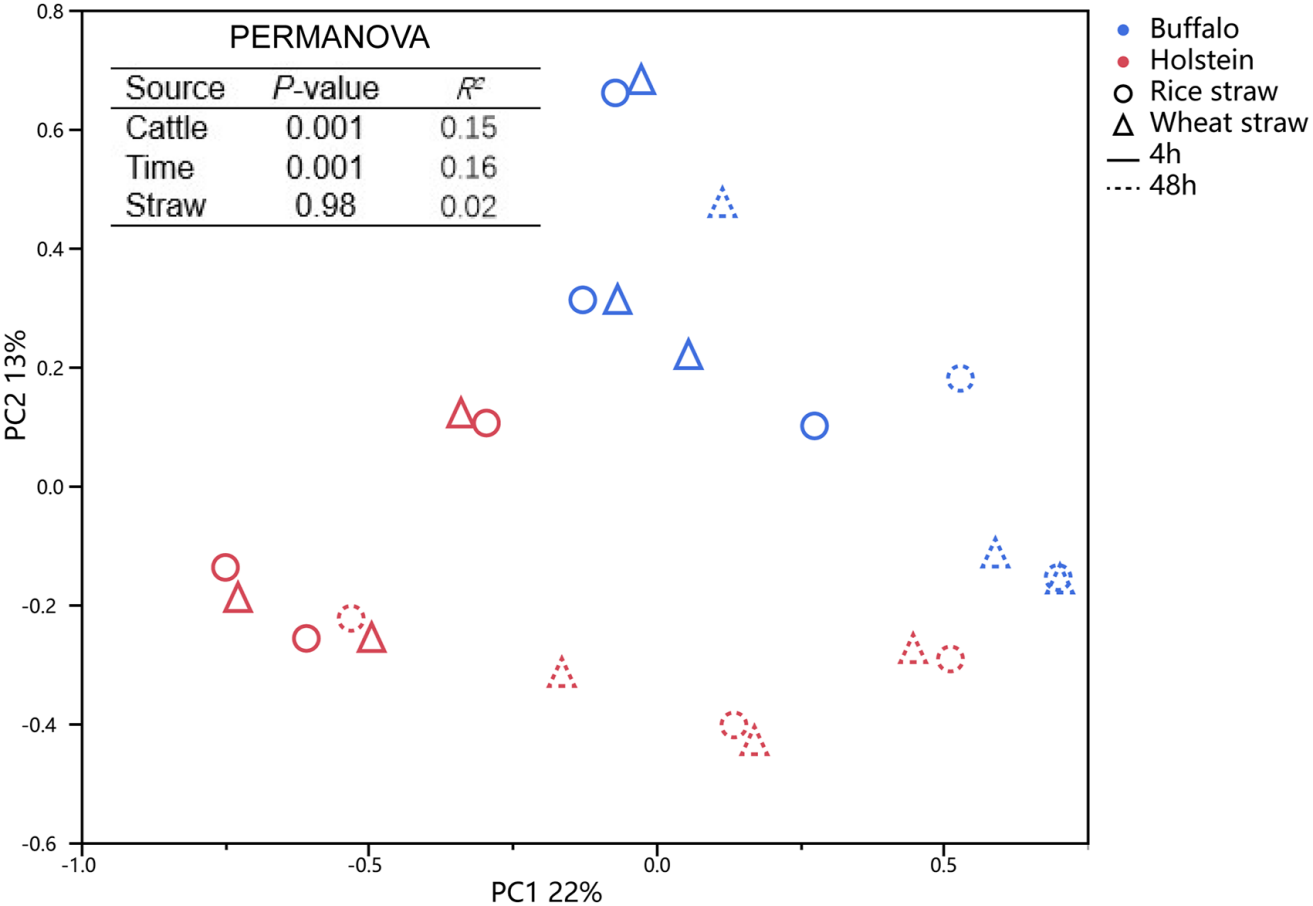
Principal coordinate analysis of bacterial community attached to rice and wheat straws at 4 and 48 h of *in situ* incubation time in buffalo and Holstein rumen. PERMANOVA, permutational multivariate analysis of variance.

The compositions of the bacteria community attached to the straw were also analyzed by 16S rRNA sequencing technique (Table 4). Although the host animal did not alter (*p* > 0.05) the observed ASV, Chao 1 value, or Shannon index, it altered (*p* ≤ 0.01) the bacterial community attached to the straw particles (Figure 2). Buffalo exhibited a greater relative abundance of *Ruminococcus* (*p* ≤ 0.05), *Roseimarinu*s (*p* ≤ 0.05), and *Lawsonibacter* (*p* ≤ 0.05), a tendency of greater *Papillibacter* (*p* ≤ 0.1), *Enterocloster* (*p* ≤ 0.1), and *Sodaliphilus* (*p* ≤ 0.1), and a tendency of lower relative abundance of *Schaedlerella* (*p* ≤ 0.1) and *Treponema* (*p* ≤ 0.1) attached to straw particles, when compared with Holstein. Prolonging incubation time increased the Shannon index (*p* ≤ 0.05) and altered (*p* ≤ 0.01) the bacterial community attached to straw particles. Greater (*p* ≤ 0.05) relative abundance of phyla Proteobacteria and Fibrobacters, and *Papillibacter*, *Monoglobus*, *Roseimarinus*, *Sodaliphilus*, *Stenotrophomonas,* and *Treponema* attached to straw particles was observed at 48 h of incubation time and with lower (*p* ≤ 0.05) phyla Bacteroidetes, and *Ruminococcus*, *Lawsonibacter*, *Paraprevotella,* and *Mucilaginibacter* than 4 h of incubation time. Wheat straw exhibited a greater (*p* ≤ 0.05) relative abundance of phyla Fibrobacters and *Papillibacter* and a lower (*p* ≤ 0.05) relative abundance of *Enterocloster* than rice straw.

**Table 4:** Relative abundance (%) of bacterial community attached to rice and wheat straws at 4 and 48 h of *in situ* incubation time in buffalo and Holstein rumen.

Items	Cattle		Time (h)		Straw		SEM	Value of *p*		
	Holstein	Buffalo	4	48	Wheat	Rice		Cattle	Time	Straw
Observed ASV	565	589	563	591	596	558	46.4	0.53	0.40	0.26
Chao 1	567	594	567	594	601	560	48.2	0.51	0.43	0.25
Shannon index	7.70	7.80	7.66	7.83	7.79	7.71	0.09	0.39	<0.01	0.14
phyla Firmicutes	43.3	44.6	43.6	44.2	43.9	43.9	3.59	0.69	0.99	0.78
*Sedimentibacter*	8.91	4.84	7.30	6.45	6.85	6.89	2.17	0.18	0.43	0.97
*Ruminococcus*	2.63	4.92	4.52	3.03	3.51	4.04	0.69	<0.01	<0.01	0.28
*Papillibacter*	1.87	2.77	1.32	3.32	2.68	1.96	0.45	0.06	<0.01	0.04
*Lawsonibacter*	1.34	1.77	1.84	1.27	1.50	1.61	0.22	0.05	<0.01	0.47
*Enterocloster*	0.61	1.72	1.23	1.10	1.00	1.34	0.42	0.08	0.32	0.02
*Desulfofarcimen*	1.29	0.86	1.12	1.03	1.09	1.06	0.27	0.22	0.51	0.87
*Monoglobus*	0.64	0.86	0.54	0.95	0.86	0.64	0.16	0.19	<0.01	0.06
*Schaedlerella*	1.36	0.79	1.13	1.02	0.96	1.19	0.31	0.08	0.62	0.30
phyla Bacteroidetes	33.3	31.5	36.9	28.0	32.8	32.0	3.87	0.52	<0.01	0.76
*Prevotella*	18.3	16.3	20.5	14.1	17.7	17.0	4.38	0.59	0.05	0.83
*Paraprevotella*	5.74	4.24	6.47	3.51	4.91	5.07	0.90	0.20	<0.01	0.74
*Roseimarinus*	0.44	1.12	0.34	1.22	0.69	0.87	0.24	0.04	<0.01	0.23
*Sodaliphilus*	0.91	2.62	0.87	2.65	1.72	1.80	0.84	0.08	<0.01	0.89
*Mucilaginibacter*	0.82	1.57	1.59	0.80	1.15	1.25	0.38	0.14	0.01	0.64
phyla Proteobacteria	14.2	15.0	11.4	17.8	13.6	15.6	2.52	0.80	<0.01	0.15
*Stenotrophomonas*	7.64	9.22	5.39	11.5	7.53	9.34	2.01	0.45	<0.01	0.17
phyla Fibrobacters	1.47	1.23	0.47	2.23	1.88	0.82	0.36	0.43	<0.01	<0.01
phyla Spirochaetes	1.26	0.71	0.74	1.23	1.22	0.75	0.39	0.07	0.10	0.11
*Treponema*	1.06	0.58	0.49	1.15	1.06	0.59	0.34	0.06	<0.01	0.07
Others	6.47	7.17	6.58	7.06	6.90	6.74	0.74	0.20	0.38	0.77
*others*	45.6	44.5	44.0	46.1	45.9	44.2	3.03	0.70	0.31	0.41

The genera with a relative abundance of <1% were grouped into “others”. SEM, standard error of mean.

## Discussion

The rumen microbial ecosystem contains abundant bacteria capable of digesting complex polysaccharides like cellulose, semi-cellulose, and lignin through microbial-mediated fermentation, which is critical to harvesting energy from feed for host animals (Mizrahi et al., 2021). The core bacteria, including phyla Firmicutes and Bacteroidetes, *Prevotella*, *Butyrivibrio*, *Ruminococcus*, unclassified *Lachnospiraceae*, *Ruminococcaceae*, *Bacteroidales*, and *Clostridiales*, comprise the majority of all bacterial sequence in the rumen ecosystem despite host range, diets, and feeding strategies (Henderson et al., 2015; Weimer, 2015). We also found that phyla Bacteroidetes, Firmicutes, and Proteobacteria, and *Prevotella*, *Paraprevotella*, *Stenotrophomonas,* and *Sphaerochaeta* were the dominant bacteria in both buffalo and Holstein.

Buffalo exhibited greater *in situ* ruminal degradation of straw than Holstein, as indicated by greater *a* and *ED* of DM and NDF, together with greater *c* of fraction *b* in our study. Xu et al. (2021) reported that buffalo exhibit greater *in situ* ruminal fiber degradation of rice straw than other cattle. *In vitro* ruminal batch culture indicates that inoculating rumen fluid from buffalo has greater NDF and ADF degradation of steam-treated sugarcane than that from Holstein cattle (Jabari et al., 2014). *In vivo* trial also indicates that buffalo exhibits greater nutrient digestibility than Hereford with a ryegrass straw diet (Hussain and Cheeke, 1996). Such enhanced fiber degradation in the buffalo rumen microbial ecosystem can be due to the enrichment of functional distinct microorganisms and enzymes involved in lignocellulose degradation (Singh et al., 2014; Zhang et al., 2017).

We then investigated the bacterial community attached to straws at the 4 and 48 h of incubation time using the 16S rRNA amplicon sequencing technique. Buffalo had a distinct bacterial community attached to straw particles as compared to Holstein, with greater 16S rRNA gene copies of bacteria and a relative abundance of *Ruminococcus*. *Ruminococcus* is one of the most predominant polysaccharides degraders, produces a set of cellulolytic enzymes like endoglucanases, exoglucanases, glucosidases, and hemicellulases (Singh et al., 2014), and plays important roles in the degradation of polysaccharides (Dai et al., 2015). Such a greater relative abundance of *Ruminococcus* could contribute to the greater *in situ* fiber degradation in buffalo.

Ruminal fiber degradation and the bacterial community attached can also be affected by the straws incubated. The rice straw had greater *b* and *ED* of DM and NDF than wheat straw, which could be caused by its lower fiber content. These results were in agreement with previous studies, which report a negative relationship between ruminal degradation and fiber content (Trujillo et al., 2010; Yari et al., 2012). Forage types can alter the microbial composition and community attached, resulting in different ruminal degradation of forages. Liu et al. (2016) reported that incubation of rice straw and alfalfa hay exhibited distinct bacterial community structures, with a greater abundance of phyla Fibrobacter, Treponema, and unclassified Bacteroidales attached to rice straw. However, this was not the case in the current study. We found that the bacterial community attached to wheat straw showed an indistinct separation by comparing with that attached to rice straw and exhibited a greater abundance of phyla Fibrobacters and 16S rRNA gene copies of *Fibrobacter succinogenes*. Both *Fibrobacter succinogenes* and phyla Fibrobacters are the main cellulose-degrading bacteria that play a critical role in degrading fiber (Ransom-Jones et al., 2012). Enrichment of cellulose-degrading bacteria did not cause an increase in ruminal fiber degradation of wheat straw in our study, which needs further investigation.

Ruminal carbohydrate degradation in the rumen occurs through a consortium of microbes that function together in a synergistic manner, and the changes in microbial community colonization with prolonged incubation time could be associated with the change in forage composition and the function of microorganisms (Piao et al., 2014; Huws et al., 2016; Cheng et al., 2017; Zhang et al., 2020). In our study, the 4-h incubation time had a greater abundance of phyla Bacteroidetes and *Prevotella* attached to the straw. Both phyla Bacteroidetes and *Prevotella* own the function of mobilizing easily accessible nutrients like soluble sugar and protein and can be more likely to colonize at the early stage of fermentation. Zhang et al. (2020) also found that the 4-h incubation time exhibits a greater abundance of phyla Bacteroidetes. Prolonging incubation time could alter the bacterial community through the increasing abundance of phyla Firmicutes and Fibrobacteres attached to straw particles (Liu et al., 2016; Zhang et al., 2020). Taxa within the phyla Firmicutes and Fibrobacteres are known to degrade the more recalcitrant polysaccharides of the plant cell wall (Sha et al., 2020). The 48-h incubation time had a greater abundance of phyla Proteobacteria and Fibrobacteres attached to the straw, which was consistent with greater fiber degradation at the later fermentation stage. It seems that prolonging incubation time promotes straw fiber degradation by enriching bacteria, and phyla Proteobacteria and Fibrobacteres colonization in straw.

## Conclusion

Buffalo exhibit greater *in situ* disappearance and effective ruminal degradation of straw fiber than Holstein, which is accompanied by the distinct bacterial community attached to straw with an increased bacteria population and relative abundance of Ruminococcus. Prolonging incubation time enriches phyla Proteobacteria and Fibrobacteres, leading to enhanced fiber degradation at the late stage of incubation, indicating that incubation time also alters the bacterial community attached to the straw.

## Data availability statement

The datasets presented in this study can be found in online repositories. The names of the repository/repositories and accession number(s) can be found at: https://www.ncbi.nlm.nih.gov/, bioproject/PRJNA893085.

## Ethics statement

The animal study was reviewed and approved by Animal Care Committee, Institute of Subtropical Agriculture, the Chinese Academy of Sciences, Changsha, China.

## Author contributions

XP and MW conceived this study. MW, ZT, BL, and BT managed the project. XP and MZ conducted this experiment. XP, XZ, QL, RW, and SZ carried out the statistical analysis. XP wrote this manuscript. MW and ZT critically reviewed the manuscript. All authors contributed to the article and approved the submitted version.

## Funding

This study was supported by the Strategic Priority Research Program of the Chinese Academy of Sciences (grant no. XDA26040203), the National Natural Science Foundation of China (grant nos. 31922080, 32002204 and 32161143028), the Hunan Province Science and Technology Plan (2020NK2066 and 2022NK2021), the Science and Technology Innovation Program of Hunan Province (2021RC2102), the China Agriculture Research System (CARS-35), and the Open Fund of Key Laboratory of Agro-ecological Processes in Subtropical Region Chinese Academy of Sciences (grant no. ISA2021203).

## Conflict of interest

The authors declare that the research was conducted in the absence of any commercial or financial relationships that could be construed as a potential conflict of interest.

## Publisher’s note

All claims expressed in this article are solely those of the authors and do not necessarily represent those of their affiliated organizations, or those of the publisher, the editors and the reviewers. Any product that may be evaluated in this article, or claim that may be made by its manufacturer, is not guaranteed or endorsed by the publisher.

## Supplementary material

The Supplementary material for this article can be found online at: https://www.frontiersin.org/articles/10.3389/fmicb.2022.1079056/full#supplementary-material

## Footnotes

^1^ http://rdp.cme.msu.edu/
