# Supplementary material for: Comparison of in situ ruminal straw fiber degradation and bacterial community between buffalo and Holstein fed with high-roughage diet
Source: Front Microbiol. 2023 Jan 9;13:1079056. doi: 10.3389/fmicb.2022.1079056 (PMC9868309; doi:10.3389/fmicb.2022.1079056)
Supplement: Supplementary file 1 [file Table_1.DOCX]

**Table S1.** The ingredients and nutrient composition of the basal diet

| Items | Value |
| --- | --- |
| Ingredients, g/kg of DM |  |
| Wheat straw | 800 |
| Corn | 116 |
| Soybean meal | 13.3 |
| Cotton meal | 15.2 |
| Wheat Skin | 15.2 |
| Soybean skin | 11.4 |
| DDGS | 9.50 |
| Urea | 10.0 |
| NaHCO_3_ | 1.90 |
| NaCl | 1.90 |
| Premix^a^ | 5.70 |
| Nutrient composition, g/kg DM |  |
| DM | 921 |
| OM | 902 |
| CP | 81.7 |
| NDF | 634 |
| ADF | 377 |

^a^Premix provided per kilogram of concentrate: 1,000,000 IU of vitamin A, 200,000 IU of vitamin D, 1,250 IU of vitamin E, 8,000 mg of Zn, 80 mg of Se, 120 mg of I, 2,000 mg of Fe, 40 mg of Co, 2,500 mg of Mn, and 2,000 mg of Cu.

DM, dry matter; OM, organic matter; CP, crude protein; NDF, neutral detergent fiber; ADF, acid detergent fiber.

**Table S2.** Primers for quantitative real-time PCR (qPCR) of microbial groups

| Microbial group | Primer | Sequence(5’-3’) | Product size | Reference |
| --- | --- | --- | --- | --- |
| Bacteria | Forward | CGGCAACGAGCGCAACCC | 146 | Denman and McSweeney., (2006) |
|  | Reverse | CCATTGTAGCACGTGTGTAGCC |  |  |
| *Fibrobacter succinogenes* | Forward | GTTCGGAATTACTGGGCGTAAA | 121 |  |
|  | Reverse | CGCCTGCCCCTGAACTATC |  |  |
| *Selenomonas ruminantium* | Forward | CAATAAGCATTCCGCCTGGG | 138 | Stevenson and Weimer., (2007) |
|  | Reverse | TTCACTCAATGTCAAGCCCTGG |  |  |
| *Prevotella.spp* | Forward | GGTTCTGAGAGGAAGGTCCCC | 121 |  |
|  | Reverse | TCCTGCACGCTACTTGGCTG |  |  |
| *Ruminococcus amylophilus* | Forward | CAATAAGCATTCCGCCTGGG | 102 |  |
|  | Reverse | TTCACTCAATGTCAAGCCCTGG |  |  |
